# Validity of treadmill- and track-based individual calibration methods for estimating free-living walking speed and VO_2_ using the Actigraph accelerometer

**DOI:** 10.1186/s13102-015-0024-7

**Published:** 2015-11-25

**Authors:** Anthony Barnett, Ester Cerin, Corneel Vandelanotte, Aya Matsumoto, David Jenkins

**Affiliations:** 1 School of Exercise and Nutrition Sciences, Deakin University, 221 Burwood Highway, Burwood, VIC 3125 Australia; 2 Institute for Health & Ageing, Australian Catholic University, Melbourne, VIC 3000 Australia; 3 School of Public Health, The University of Hong Kong, Pokfulam, Hong Kong SAR; 4 Centre for Physical Activity Studies, Central Queensland University, Rockhampton, QLD 4702 Australia; 5 Baker IDI Heart and Diabetes Institute, 75 Commercial Road, Melbourne, VIC 3004 Australia; 6 School of Human Movement and Nutrition Sciences, The University of Queensland, Brisbane, QLD 4072 Australia

**Keywords:** Wearable devices, Energy expenditure, GPS, Physical activity, Exercise, Measurement

## Abstract

**Background:**

For many patients clinical prescription of walking will be beneficial to health and accelerometers can be used to monitor their walking intensity, frequency and duration over many days. Walking intensity should include establishment of individual specific accelerometer count, walking speed and energy expenditure (VO_2_) relationships and this can be achieved using a walking protocol on a treadmill or overground. However, differences in gait mechanics during treadmill compared to overground walking may result in inaccurate estimations of free-living walking speed and VO_2_. The aims of this study were to compare the validity of track- and treadmill-based calibration methods for estimating free-living level walking speed and VO_2_ and to explain between-method differences in accuracy of estimation.

**Methods:**

Fifty healthy adults [32 women and 18 men; mean (SD): 40 (13) years] walked at four pre-determined speeds on an outdoor track and a treadmill, and completed three 1-km self-paced level walks while wearing an Actigraph monitor and a mobile oxygen analyser. Speed- and VO_2_-to-Actigraph count individual calibration equations were computed for each calibration method. Between-method differences in calibration equation parameters, prediction errors, and relationships of walking speed with VO_2_ and Actigraph counts were assessed.

**Results:**

The treadmill-calibration equation overestimated free-living walking speed (on average, by 0.7 km · h^−1^) and VO_2_ (by 4.99 ml · kg^−1^ · min^−1^), while the track-calibration equation did not. This was because treadmill walking, from which the calibration equation was derived, produced lower Actigraph counts and higher VO_2_ for a given walking speed compared to walking on a track. The prediction error associated with the use of the treadmill-calibration method increased with free-living walking speed. This issue was not observed when using the track-calibration method.

**Conclusions:**

The proposed track-based individual accelerometer calibration method can provide accurate and unbiased estimates of free-living walking speed and VO_2_ from walking. The treadmill-based calibration produces calibration equations that tend to substantially overestimate both VO_2_ and speed.

## Background

Through its *Exercise is Medicine* initiative, the American College of Sports Medicine encourages regular assessment and inclusion of physical activity in treatment plans as a component of all medical care [1] and physical activity monitoring has become more common in clinical practice [2]. Clinical prescription and monitoring of post-diagnosis physical activity may benefit patients with specific disease states [3] and may be also used for morbidity prevention. Walking, a preferred and versatile form of physical activity, has been associated with decreased risks of cardiovascular disease and all-cause mortality [4, 5], decreased body fatness and resting diastolic blood pressure and increased aerobic fitness [6].

Due to between-individual variability in exercise capacity, health status and pre-intervention levels of physical activity, prescribed exercise intensity (e.g., walking speed) and duration should be determined on an individual basis. As self-reports can be influenced by poor recall and reporting bias, objective assessment is preferable when monitoring the patient during treatment [2]. Accelerometers are small, non-intrusive physical activity monitors appropriate for the clinical setting [2]. They are suited for objectively monitoring walking intensity, frequency and duration over many days. Actigraph is the most widely used brand of accelerometer [7].

Actigraph accelerometers have been extensively studied [8] and group calibration equations converting Actigraph outputs (counts per time unit) into energy expenditure (VO_2_) and physical activity intensities have been derived [9]. However, substantial between-individual variations in the relationships between Actigraph outputs, walking speed and VO_2_ have been reported [10–12]. This means that to accurately assess a patient’s VO_2_ and walking intensity, frequency and duration during treatment, determination of prescribed walking intensity should include establishment of individual specific accelerometer count, walking speed, VO_2_ relationships [13]. This assessment can be undertaken using a graded intensity protocol on a treadmill or during overground walking. However, while equivalence was found between 3 % slope treadmill and horizontal overground walking counts using an RT3 accelerometer [14], Actigraph counts have been reported to be lower during horizontal treadmill than overground walking [12]. Conversely, VO_2_ at a given walking speed has been found to be higher on a treadmill in comparison to overground walking [12, 15, 16]. Therefore, treadmill-based individual calibration may yield biased estimates of free-living level walking speed and VO_2_.

A cost-effective, simple track-based calibration method to estimate free-living level walking speed and VO_2_ using Actigraph counts has been proposed [10]. This method requires the participants to walk at four pre-determined speeds controlled by a GPS monitor while wearing an Actigraph accelerometer and a mobile oxygen analyser. The calibration procedure is of similar duration to other calibration protocols [14, 17], but does not require a treadmill nor does it require a track. The protocol can be conducted on any flat, solid unobstructed pathway. Participants are asked to walk at four different speeds to allow the estimation of quadratic individual calibration equations, since the relationship between Actigraph output and energy expenditure from walking is curvilinear [18, 19]. This method has been shown to have good reliability and validity with respect to the estimation of free-living level walking speed [10]. However, its validity for the estimation of VO_2_ from free-living level walking and how it compares to treadmill-based calibration is unknown.

The primary aim of this study was to compare the validity of the track- and treadmill-based calibration methods for estimating free-living level walking speed and VO_2_ using the Actigraph monitor. The secondary aim was to explain between-method differences in accuracy of estimation of free-living walking speed and VO_2_. To achieve the second aim, differences between calibration equations and relationships of calibration-trial walking speed with VO_2_ and Actigraph counts obtained using the two methods were assessed.

## Methods

### Participants

Fifty healthy adults (32 women and 18 men), recruited either by word of mouth or by responding to local advertisements, participated in this study. The descriptive characteristics (mean (SD)) of the male participants were age 41 (14) years, height 182 (7) cm, and weight 81 (13) kg, and of the female participants age 40 (12) years, height 167 (7) cm, and weight 63 (9) kg. All participants provided written, informed consent and completed the Physical Activity Readiness Questionnaire. The Medical Research Ethics Committee at the University of Queensland approved the study.

### Experimental protocol

Data were collected in 2006. Participants reported to the laboratory at the pre-arranged time (which varied across participants) and their height (stadiometer) and weight were measured. They then performed a treadmill walk, an outdoor track walk and a free-walk in randomized order on the same day. These walks were separated by a minimum of 10 min. For the duration of all walks VO_2_ by indirect calorimetry (mobile oxygen analyser - Cosmed K4b^2^, Cosmed, Rome, Italy) and accelerometer counts (Actigraph 7164) were recorded continuously. Clock times of the Cosmed, Actigraph and the GPS monitor (Forerunner 201, Garmin Ltd, Olathe, KS), used during the outdoor data collection, were synchronized to the time on the computer used to initialize the Actigraphs.

#### Treadmill walk (calibration trial)

Following a 5-min familiarization period and a 5-min recovery, participants walked on a calibrated treadmill (Bodyguard Cartier 312-C; Bodyguard Fitness, Quebec, Canada). The continuous protocol consisted of 5-min walks at 3.5, 4.5, 5.5 and 6.5 km · h^−1^. VO_2_ (ml · kg^−1^ · min^−1^) breath-by-breath data were collected over the last 2 min at each speed (allowing 3 min at each level to reach steady-state VO_2_) and Actigraph count (counts · min^−1^) was taken as the mean of full minute counts at each speed.

#### Outdoor track walk (calibration trial)

The walk was carried out on a 400-m track with speed controlled by an investigator walking using a Forerunner GPS monitor and the subject instructed to maintain a distance of approximately 2 m behind. The Forerunner was set on lap speed function (400 m laps) as we have previously found this most appropriate for maintaining speed [10]. The walk was continuous and comprised of 400 m at 3.5, 4.5, and 5.5 km · h^−1^, and 800 m at 6.5 km · h^−1^. VO_2_ and Actigraph count for each speed were determined in the same way as for the treadmill walk. The extra lap at 6.5 km · h^−1^ was undertaken to ensure time for adequate full-minute Actigraph counts. As each lap speed was determined separately and may vary from the protocol speed, VO_2_ at this speed was determined for the last 2 min of each lap and the speeds and VO_2_ values were then averaged.

#### Overground level walk (free-living walks)

A 3-km walk was undertaken on a flat, outdoor walking path. The walk comprised of 1-km walks at self-assessed slow, moderate and brisk pace in randomized order. To control distance and determine speed, participants wore a Forerunner with the lap function set to 1-km. At the end of each kilometer the Forerunner emitted an audible sound and the subject proceeded to the next walking pace (at 1 and 2 km) or the walk was terminated (at 3 km). Participants were asked to walk at a consistently maintained, self-paced speed without reference to the GPS display. For each 1-km walk, VO_2_ (ml · kg^−1^ · min^−1^) was estimated as the mean value from 3 min until the end point of the walk to ensure steady state values. Accelerometer counts during walking have some variation, even at a constant speed on a treadmill. To ensure the most representative count for that speed, Actigraph count was estimated as the mean of all full-minute counts during the 1 km. The minute counts at the transition from one speed to the next were excluded from the analysis.

#### Protocol without indirect calorimetry

To examine the possible effects of wearing a gas analyzer on the relationship between Actigraph counts and walking speed through alterations of walking patterns, a group of participants (*n* = 15) repeated the entire protocol without wearing the Cosmed.

### Materials

#### GPS monitor

The Forerunner 201 GPS monitor was used to control calibration-trial walking speed and record distance and speed for the track walks. It was also used to control distance and record walking speed during the overground, free-living walks. GPS monitors determine speed by Doppler shift: the rate of change of satellite radio frequency signals resulting from movement of the receiver [20]. The Forerunner is designed to be worn on the wrist. We have previously found Forerunner and chronometer determined speeds during 400-m track walks (*n* = 40) to be virtually identical (*R*^2^ = 0.999, slope = 1.001 and the intercept not significantly different from zero) [10]. Following each data collection period the Forerunner data were downloaded (Forerunner Logbook Version 2.5, Garmin Ltd, Olathe KS) to determine actual speeds for each trial component.

#### Actigraph accelerometer

The Actigraph 7164 is a uniaxial accelerometer and has been described in detail elsewhere [21]. Prior to each data collection session, the calibration of the Actigraph 7164 accelerometer was checked according to the manufacturer’s instructions using the Actigraph Calibrator CAL71. All readings over the duration of the study were within the manufacturer’s limits. The Actigraph was initialized using 1-min epochs. For the duration of each testing session, the Actigraph was worn against the skin at waist level on the right anterior-axillary line and held in place by a firmly-fitted elasticized belt. The Actigraph data were downloaded to a PC and the mean full-minute counts were determined for each speed in all trials.

#### Indirect calorimetry (VO_2_)

Prior to each data collection session the mobile oxygen analyser was calibrated according to the manufacturer’s instructions. A room air calibration and a reference calibration (15.1 % O_2_ and 5.15 % CO_2_) were undertaken to calibrate the oxygen and carbon dioxide analyzers. A 3-l syringe (Hans-Rudolf) was used to calibrate the flow turbine and a delay calibration was carried out to adjust for lag time between the expiratory flow and gas measurements. Ambient air calibrations were also undertaken prior to each trial. Breath-by-breath data were collected continuously during all trials and the mean VO_2_ (ml · kg^−1^ · min^−1^) for the relevant time period for each trial was determined. VO_2_ data were adjusted for the mass of the equipment worn (1.2 kg).

### Statistical analysis

Average full-minute Actigraph counts · min^−1^, walking speed (km · h^−1^), and VO_2_ (ml · kg^−1^ · min^−1^) were computed for all walks, at all speeds in all participants.

#### Determination of individual calibration equations

To obtain individual calibration equations, ordinary-least-squares (OLS) linear regression models were used. Previous studies have found a linear relationship between calibration-trial walking speed and accelerometer counts [8, 10]. Consequently, to estimate individual calibration equations, calibration-trial walking speed (four walking speeds per calibration method per subject) was regressed onto Actigraph counts. This resulted in two walking speed-Actigraph counts individual calibration equations per subject, one for each calibration method. Each of these calibration equations yielded an intercept and a slope (regression coefficient) for each subject. The intercept value represented the estimated walking speed at 0 Actigraph counts · min^−1^, while the slope represented the estimated increase in walking speed associated with a 1 unit increase in Actigraph counts · min^−1^ for a particular subject for a particular calibration method. As a curvilinear relationship between accelerometer counts and VO_2_ has been reported [22], VO_2_ was regressed onto Actigraph counts (linear) and their squared value (quadratic term). Two VO_2_-Actigraph counts individual calibration equations per subject were obtained, one for each calibration method. Each of these calibration equations yielded an intercept and two slopes for each subject. The intercept value represented the estimated VO_2_ at 0 Actigraph counts · min^−1^, while the sum of the two slopes represented the estimated increase in VO_2_ associated with a 1 unit increase in Actigraph counts · min^−1^ for a particular subject for a particular calibration method. The fit of linear and quadratic regression equations to the data were compared by examining their coefficients of determination (*R*^2^).

#### Determination and comparison of validity of treadmill- and track-based calibration equations (primary study aim)

To examine and compare the validity of the two individual calibration methods for estimating free-living walking speed and VO_2_ using Actigraph counts, differences between expected (estimated using the individual calibration equations) and actual (measured) values of speed and VO_2_ collected during 1-km self-paced (free-living) walks were computed (here named prediction errors; Δ). The average prediction error for a calibration method represents its systematic bias of prediction. A value significantly greater than 0 indicates positive bias (overestimation of speed or VO_2_), while a value significantly smaller than zero indicates negative bias (underestimation of speed or VO_2_). Linear mixed models with random intercepts and slopes were then used to estimate: 1) the prediction errors for each calibration method for the ‘average’ subject walking at the ‘average’ speed (obtained by centering free-living walking speed around the group mean) on the free-living walks; 2) the between-participant (i.e., between-subject) variability in prediction errors; and 3) the extent to which the magnitude of the prediction errors (linearly or quadratically, based on an inspection of scatter plots with lowess smoother lines) depended on actual free-living walking speed.

Linear mixed models were used because the assumption of independency of observations was violated. Specifically, there were three sets of data points per subject, one for each free-living walk (participants undertook three 1-km self-paced walks). Thus, in order to obtain correct standard errors of regression coefficients, we needed to use models that take into account the existing correlation among data collected from the same participants. Linear mixed models with random intercepts and slopes were also considered appropriate in the context of this study because they fit the notion of person-specific effects implied by individual calibration by allowing intercept and slopes to vary across participants. They provide estimates of the values of the intercept and slopes for the average participant and also provide information on the variability of these across participants. Parameters were estimated using the residual maximum likelihood method (REML). The Aikake Information Criterion (AIC) and a modified likelihood ratio test [23] were used to test the significance of the between-subject variability in regression parameters. Additionally, standard errors of estimate (here called total error; TE) were computed using the formula: TE = [∑(*Y* − *Y*′)^2^/(*N* − 1)]^0.5^, where *Y* is the measured value of speed or VO_2_ during a free walk, *Y*′ is the estimated value of speed or VO_2_ computed using the individual calibration equations and *N* is the number of observations. Total error represents the standard error of estimate obtained when an existing equation (e.g., an individual calibration equation) is cross-validated (i.e., its accuracy in predicting the newly collected data is evaluated) [24].

#### Differences between calibration methods (secondary study aim)

##### Between-method differences in individual calibration equations

Paired *t*-tests were used to test the significance of the differences in OLS slopes and intercepts between the treadmill and track individual calibration equations described above for each outcome (speed and VO_2_). Paired *t*-tests were used because estimates of two calibration equations obtained on the same participants were compared. While the relationship between Actigraph counts and speed was linear and quantified by one slope, the relationship between Actigraph counts and VO_2_ was quadratic and quantified with two slopes (one for the linear and the other for quadratic term). Consequently, for VO_2_, between-method differences were tested for the sum of the slopes of the linear and quadratic terms of Actigraph counts (representing the ‘effect’ of Actigraph counts). Also, *t*-tests were used to test the significance of the between-method difference in expected values of speed and VO_2_ (as predicted by the respective individual calibration equations) at tertiles of Actigraph counts. The tertile values of Actipraph counts per minute were determined from data collected during the calibration trials.

##### Between-method differences in relationships of calibration-trial walking speed with Actigraph counts and VO_2_

Linear mixed models with random intercepts and slopes were used to account for dependency in the observations (multiple data points per subject) and the fact that the relationships of interest vary across individuals. Actigraph counts · min^−1^ were modeled as a function of the linear term of calibration-trial walking speed centred at 3.5 km · h^−1^ (the lowest walking speed used), the calibration method (treadmill vs. track), and the interaction term of calibration-trial walking speed and calibration method, while VO_2_ was modeled as a function of the linear and quadratic terms of speed, the calibration method, and the interaction terms of the calibration method and the linear and quadratic terms of speed. Between-participant variation in regression coefficients was tested using the modified likelihood ratio test and by comparing AIC values. Parameter estimation was performed using the REML method. However, to evaluate the significance of interaction terms, models with and without interaction terms were compared using AIC values estimated with the maximum likelihood method.

Six additional sets of models as those described above but each with speed centered at one of the three remaining calibration-trial walking speeds (4.5, 5.5, and 6.5 km · h^−1^) and with different calibration-method reference categories (treadmill vs. track) were constructed. They were used to obtain estimates of the mean Actigraph counts and VO_2_ (and associated standard errors and between-individual variability measures) at each calibration-trial walking speed and for each calibration method.

#### Differences in relationships between Actigraph counts and walking speed when wearing and not wearing the mobile oxygen analyser

Linear mixed models with random intercepts and slopes were used. A model for each component of the experimental protocol (i.e., treadmill-based calibration; track-based calibration; and free-living walks) was constructed to test the differences in calibration-trial and free-living walking speed-Actigraph counts relationship when wearing and not wearing the mobile oxygen analyser. Actigraph counts · min^−1^ were modeled as a function of the linear term of walking speed, the experimental condition (with or without the mobile oxygen analyser), and the interaction term of walking speed and experimental condition. Non-significant interaction terms were omitted from the models to re-estimate main effects. Speed was centered at 3.5 km · h^−1^. All slopes and intercepts were allowed to vary across participants. All analyses were conducted using Stata 9.0 (StataCorp) and MLwiN 2.02 (Multilevel Models Project).

## Results

### Determination of individual calibration equations

A linear relationship between Actigraph counts and speed of walking during the calibration trials was supported by individual scatter plots with superimposed fitted lines. Support was found for the hypothesis of a quadratic relationship between Actigraph counts and VO_2_ from walking. The individual *R*^2^ values from the quadratic regression models ranged from 0.91 to 1.00 (mean = 0.99). In contrast, the individual *R*^2^ values from linear regression models ranged from 0.84 to 1.00 (mean = 0.95).

### Determination and comparison of validity of treadmill- and track-based calibration equations (primary study aim)

**Fig. 1:**
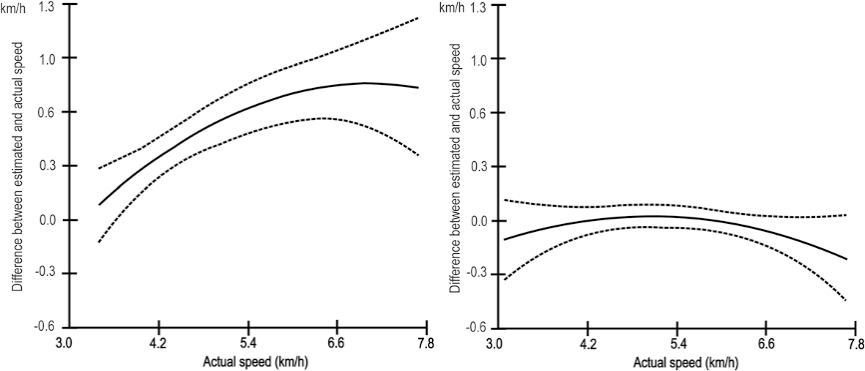
Prediction error of walking speed on the free-living walks for treadmill- and track-based calibration methods. Figure 1 shows the relationship between mean prediction error for walking speed (km · h^−1^) and actual walking speed (km · h^−1^) on the free-living walks for the treadmill- (*left*) and track-based (*right*) calibration methods. *Dashed lines* are 95 % confidence intervals

Overall, the track-based individual calibration equations estimated free-living walking speed and VO_2_ consumption from Actigraph counts more accurately than did the treadmill-based calibration equations (Table 1). The treadmill method tended to overestimate speed as well as VO_2_ (see intercept values in Table 1). For example, for the ‘average’ subject walking at a speed of 5.7 km · h^−^1, which was the average speed of free-living walks across all individuals, the treadmill-based calibration equation overestimated actual speed by 0.7 km · h^−1^ (95 % CI: 0.56, 0.87 km · h^−1^). The average magnitude of the prediction error depended on the actual speed of walking (Fig. 1 and slopes for speed and speed^2^ in Table 1). In contrast, the track-based calibration equation did not tend to yield biased estimates of speed for the average subject (Fig. 1) and the prediction error did not depend on actual free-living walking speed. Additionally, the between-individual variations in prediction error across values of actual free-living walking speed were smaller for the track-based than the treadmill-based method (smaller between-individual variability of intercept and slopes) (Table 1).

**Table 1:** Regression models of prediction error for walking speed and VO_2_ by calibration method

	Prediction error for speed of walking (km · h^−1^)		Prediction error for VO_2_ (ml · kg^−1^ · min^−1^)	
Regression parameter	Treadmill method	Track method	Treadmill method	Track method
	Coefficient (95 % CI ) BPV	Coefficient (95 % CI) BPV	Coefficient (95 % CI) BPV	Coefficient (95 % CI) BPV
Intercept	0.71*** (0.56, 0.87) 0.54***	0.02 (−0.05, 0.08) 0.19***	4.99*** (3.96, 6.00) 3.26***	0.33 (−0.06, 0.73) 1.10***
Speed	0.15*** (0.06, 0.24) 0.26***	−0.04 (−0.09, 0.01) 0.08*	1.16*** (0.58, 1.75) 1.57***	−0.29** (−0.49, −0.08) –
(Speed)^2^	−0.06* (−0.11, −0.01) 0.13*	−0.04 (−0.09, 0.01) 0.10**	– – –	−0.20* (−0.38, −0.02) –

These regression models report on average prediction error by calibration method (see intercept values) and if this error depends on walking speed (see regression coefficients for speed and speed^2^). Coefficient = regression coefficient; 95 % CI = 95 % confidence interval; BPV = Between-participant variability in regression coefficients (expressed as standard deviations); – = not applicable (not significant regression term or between-participant variability based on a comparison of fixed and random slope models); *p < .05; **p < .01; ***p < .001. Speed (km · h^−1^) was centered at 5 · 7 km.h^−1^

**Fig. 2:**
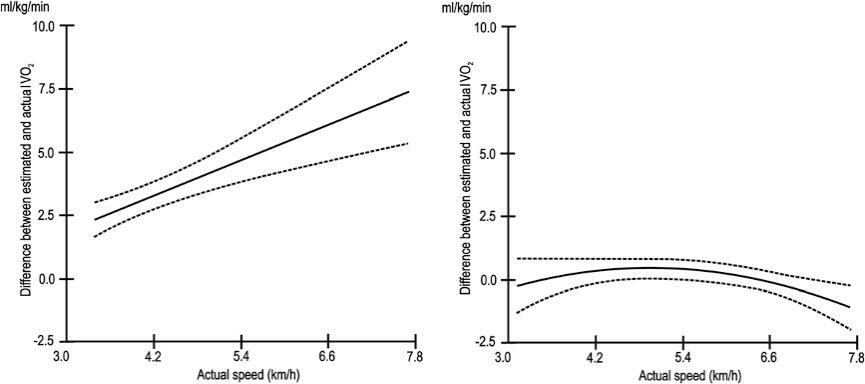
Prediction error of VO_2_ on the free-living walks for the treadmill- and track-based calibration methods. Figure 2 shows the relationship between mean prediction error for VO_2_ (ml · kg^−1^ · min^−1^) and actual walking speed (km · h^−1^) on the free-living walks for the treadmill- (*left*) and track-based (*right*) calibration methods. *Dashed lines* are 95 % confidence intervals

For the average subject walking at 5.7 km · h^−1^, the treadmill-based calibration equations overestimated VO_2_ on average by 4.99 ml · kg^−1^ · min^−1^ (95 % CI: 3.97, 6.01 ml · kg^−1^ · min^−1^; Table 1), which corresponds to ~ 1.43 METs (units of metabolic equivalent). The magnitude of the prediction error increased with an increase in actual free-living walking speed (Fig. 2 and slopes for speed and speed^2^ in Table 1). No significant bias was observed for the track-based calibration method. The prediction error appeared to be curvilinearly related to actual speed of walking (slopes for speed and speed^2^ in Table 1 and Fig. 2). However, when free-living walking speeds outside of the range of speeds used to derive the quadratic calibration equations for VO_2_ (3.3 km · h^−1^ > speed > 6.7 km · h^−1^) were excluded from the model, the effect of speed on the prediction error for VO_2_ was not significantly different from zero (b_speed_ = −0.01, 95 % CI; −0.36, 0.33; b_speed^2_ = 0.05, 95 % CI; −0.26, −0.40). The between-participant variability in prediction error for VO_2_ was larger for the treadmill than the track calibration method (between-participant variability of intercepts: 3.28 vs. 1.10; see Table 1).

The total error (TE) for the estimation of free-living walking speed from Actigraph counts was 0.9 km · h^−1^ for the treadmill calibration method and 0.3 km · h^−1^ for the track method. The TE for the estimation of VO_2_ from Actigraph counts was 6.11 ml · kg^−1^ · min^−1^ for the treadmill and 1.50 ml · kg^−1^ · min^−1^ for the track method. When limiting the computation to values of free-living walking speed that were within the ranges of values used for deriving the calibration trials the TE decreased to 5.49 ml · kg^−1^ · min^−1^ and 1.45 ml · kg^−1^ · min^−1^, respectively. These estimates need to be interpreted with care, especially those for the treadmill method, since the prediction error was dependent on free-living walking speed (Table 1).

### Differences between calibration methods (secondary study aim)

#### Between-method difference in calibration equations

For given tertile values of Actigraph counts per minute (within the examined range of calibration-trial walking speeds), the treadmill method tended to give higher estimates of speed than the track method (Table 2). This was due to higher average slope values for the calibration equations based on the treadmill method (see slopes of Actigraph counts in Table 2). Significant between-method differences in intercepts were found for the calibration equations of VO_2_ with the treadmill method yielding greater values of VO_2_ than the track calibration method (Table 2). When examining the differences in estimated VO_2_ at tertiles of Actigraph counts, it was found that the treadmill method was associated with higher estimates of VO_2_. These differences ranged from 2.85 to 4.79 ml · kg^−1^ · min^−1^ of oxygen (see estimated speed or VO_2_ at specific Actigraph counts in Table 2).

**Table 2:** Differences in speed and VO_2_ calibration regression equation estimates between treadmill- and track-calibration methods

Regression estimates	Speed (km · h^−1^)	VO_2_ (ml · kg^−1^ · min^−1^)
	Mean ∆ treadmill – track (95 % CI)	Mean ∆ treadmill – track (95 % CI)
Intercepts of calibration equations	−0.02 (−0.16, 0.12)	2.00 (1.18, 2.82)***
Slopes of Actigraph counts^a^	1.5·10^−4^ (1.1·10^−4^, 1.9·10^−4^)***	2.9·10^−4^ (4.1·10^−4^, 9.8·10^−4^)
Estimated speed or VO_2_ at specific Actigraph counts		
… 2000 counts · min^−1^	0.29 (0.19, 0.39)***	2.85 (0.42, 3.28)***
… 3300 counts · min^−1^	0.49 (0.37, 0.61)***	3.71 (3.18, 4.24)***
… 4600 counts · min^−1^	0.70 (0.54, 0.86)***	4.79 (3.87, 5.71)***

^a^While the relationship between Actigraph counts and speed was linear and quantified by one slope, the relationship between Actigraph counts and VO_2_ was quadratic and quantified with two slopes (one for the linear and the other for quadratic term). Consequently, for VO_2_, between-method differences were tested for the sum of the slopes of the linear and quadratic terms of Actigraph counts (representing the total ‘effect’ of Actigraph counts). ∆ treadmill – track = difference between regression estimates of treadmill and track calibration equations; CI = confidence interval; ***p < .001

#### Between-method differences in relationships of calibration-trial walking speed with Actigraph counts and VO_2_

Walking on a treadmill was associated with higher levels of VO_2_ than walking on a track at a given speed (Tables 3 and 4). For the average subject walking at 3.5 km · h^−1^, overground walking was associated with a 2.25 (95 % CI: −2.62, −1.89) ml · kg^−1^ · min^−1^ lower VO_2_ as compared to walking on the treadmill (see regression coefficient for C. method in Table 3). This difference tended to remain stable across walking speeds (see regression coefficient for C. method by Speed in Table 3). Walking on a treadmill was associated with lower Actigraph counts than overground walking on a track. The average subject walking at 3.5 km · h^−1^ had 262 (95 % CI: 158, 366) more counts per minute when walking on a track than when walking on a treadmill (Table 4). This between-method difference tended to increase by 198 (95 % CI: 147, 249) counts · min^−1^ for each km · h^−1^ increase in speed. Between-participant variations in the between-method difference in VO_2_ and Actigraph counts were observed (see BPV in Tables 3 and 4).

**Table 3:** Differences between calibration methods in regression models of Actigraph counts and VO_2_ predicted by walking speed

	Outcome: Actigraph counts · min^−1^		Outcome: VO_2_ (ml · kg^−1^ · min^−1^)	
Parameter	Coef IPV	(95 % CI)	Coef IPV	(95 % CI)
Intercept	1442*** 418***	(1319, 1565)	8.96*** 2.13***	(8.37, 9.55)
Speed (centred at 3.5 km.h^−1^)	1134*** 247***	(1061, 1207)	1.56*** 0.61**	(1.28, 1.84)
(Speed)^2^	– –	–	0.30*** 0.22***	(0.21, 0.40)
C. method	262*** 302***	(158, 366)	−2.25*** 1.10***	(−2.62, −1.89)
C. method × Speed	198*** 145***	(147, 249)	−0.33 0.37*	(−0.68, 0.02)
C. method × (Speed)^2^	– –	–	0.08 0.12*	(−0.03, 0.19)

These regression models report on differences between calibration methods in Actigraph counts and VO_2_ at 3.5 km.h^−1^walking speed (see regression coefficients for C. method), and differences between calibration methods in associations of walking speed with Actigraph counts and VO_2_ (see regression coefficients for C. method by Speed and C. method by (Speed^2^)). Coef = regression coefficient; 95 % CI = 95 % confidence interval; BPV = between-participant variability in regression coefficients (expressed as standard deviations); – = not applicable; C. method = calibration method (treadmill is reference category); ^*^p < .05; ^**^p < .01; ^***^p < .001

**Table 4:** Actigraph counts and VO_2_ at given walking speeds by calibration method

	Actigraph counts · min^−1^				VO_2_ (ml · kg^−1^ · min^−1^)			
	Treadmill calibration		Track calibration		Treadmill calibration		Track calibration	
Speed (km · h^−1^)	mean (SE)	BPV	mean (SE)	BPV	mean (SE)	BPV	mean (SE)	BPV
3.5	1442 (63)	418	1704 (58)	454	8.96 (0.30)	2.32	6.86 (0.28)	2.38
4.5	2567 (78)	515	3036 (75)	556	10.62 (0.33)	2.73	8.47 (0.30)	2.73
5.5	3711 (199)	692	4368 (102)	741	13.44 (0.38)	3.80	10.86 (0.35)	3.80
6.5	4846 (136)	902	5700 (132)	963	16.53 (0.47)	5.75	14.02 (0.44)	6.35

Mean = estimated mean outcome value at specific walking speed; SE = standard error of the estimated mean; BPV = Between-participant variability around the mean (expressed as standard deviations). These estimates were obtained from models similar to those presented in Table 3 with speed centered at the various calibration-trial walking speeds (3.5, 4.5, 5.5, and 6.5 km.h^−1^) and with a different calibration-method reference category

#### Differences in relationships between Actigraph counts and calibration-trial and free-living walking speeds when wearing and not wearing the mobile oxygen analyser

No significant main effects of experimental condition (i.e., wearing and not wearing the mobile oxygen analyser) on Actigraph counts were found (all *p*-values > 0.13; in models without experimental condition by speed of walking interaction terms). Also, no significant interaction effects of experimental condition and speed of walking on Actigraph counts were observed (all *p*-values > 0.48). Finally, the data did not give sufficient support for the presence of between-individual variations in these effects.

## Discussion

The primary aim of this study was to compare the validity of a new track-based and a traditional treadmill-based calibration method for estimating free-living level walking speed and VO_2_ using the Actigraph monitor. The treadmill-based method tended to overestimate speed and, especially, VO_2_ while the overground method did not. These differences were due to treadmill walking being associated with higher VO_2_ and lower Actigraph counts than overground walking for a given walking speed. The average magnitude of overestimation of VO_2_ (for the treadmill method) at the average free-living walking speed of 5.7 km · h^−1^ corresponded to approximately 1.4 METs. The average model prediction error increased at higher speeds of walking by ~ 0.33 METs per km · h^−1^. The model prediction error for VO_2_ varied across individuals and, for the average free-living walking speed, was (mean ± SD) 4.99 ± 3.28 ml · kg^−1^ · min^−1^. This implies that the treadmill-based calibration method might overestimate energy expenditure from brisk level walking by more than 2 METs in 20 % of the population represented by the study sample. According to the data from this study, the average overestimation of energy expenditure for slow pace walking (up to 4 km · h^−1^) would be approximately 0.86 METs and that for strolling (up to 3.2 km · h^−1^) 0.60 METs. This is a substantial bias. The U.S. Department of Health and Human Services physical activity recommendations advocate at least 150 min of moderate-intensity physical activity per week [25], corresponding to activities of 3–5.9 METs [26]. While slow level walking is usually considered light-intensity activity (2.5 METs), with a positive bias of 0.86 METs, treadmill-based calibration equations would tend to classify it into moderate-intensity activities. Overestimation of energy expenditure by treadmill-based calibration equations has been also reported by Yngve and colleagues [12]. However, their study did not specifically focus on walking and they used pooled rather than individual calibration equations.

With regards to estimation of free-living walking speed, the average prediction error associated with the treadmill-based method ranged from 0.4 to 1.0 km · h^−1^ across the examined speeds. There was considerable between-individual variability in the prediction error as well as in the effect of actual free-living walking speed on the magnitude of prediction error. In contrast, the track-based method did not produce a significant bias at the average free-living walking speed (for the average subject), and the model prediction error was not significantly related to free-living walking speed. The results of this study, hence, give support for the hypothesis that treadmill walking is sufficiently different from overground walking in both biomechanical characteristics and energy economy to raise concerns about its utility for the estimation of free-living walking from accelerometer counts.

The secondary aim of this study was to explain differences in estimation of free-living walking speed and VO_2_ between the two calibration methods. Lower Actigraph counts and higher VO_2_ at a given speed during treadmill as compared to overground walking were found. These may be explained by reported differences in gait parameters during treadmill and overground walking. Most studies have found higher cadence and shorter stride length during treadmill walking [16, 27–30]. Reducing stride length at a given speed is associated with decreased vertical centre of mass displacement (flatter trajectory) and increased energy cost of walking [31, 32]. The flatter trajectory would be expected to result in lower accelerometer counts and a negative relationship between Actigraph counts and cadence has been reported [33].

In the present study, the total energy cost of walking on a treadmill was 33 % higher than that of walking on a track at 3.5 km · h^−1^. The proportional difference in energy cost between treadmill and overground walking decreased at higher walking speeds. This is in accordance with the finding that the economy of work production by muscles in flat-trajectory walking increased linearly with walking speed, while that of normal walking plateaus at a speed of around 4.5 km · h^−1^ [32].

Substantial between-individual variations were observed in intercepts and slopes of the calibration equations (Table 2). Additionally, between-individual differences were also found in the average prediction error of free-living level walking speed and VO_2_. These between-individual differences were substantially greater for the treadmill- than the track-based calibration equations. This might be due to the fact that overground walking patterns are significantly more variable across individuals than are treadmill walking patterns. In this respect, Warabi and colleagues [28] observed greater between-individual variability in stance period, heel contact time and ratio of rear foot phase over stance period in overground, level walking than treadmill walking. This means that treadmill-based calibration equations cannot capture a significant portion of between-individual variability in walking patterns, while track-based calibration equations can. This explains why the between-individual variability in model prediction error from track-based equations were less than half those from the treadmill-based calibration equations.

### Study limitations

The track-based calibration method validated in this study is limited in the sense that it can accurately work only for overground walking on flat ground. It is obvious that calibration equations obtained with this method will produce negatively biased estimates of VO_2_ for graded walking or walking whilst carrying a weight. However, most walking appears to take place on flat ground [34]. Importantly, sedentary individuals, who are the principal target of public health initiatives, would seem more likely to adopt level than graded walking as a form of activity. Consequently, this calibration method has the potential to accurately estimate VO_2_ from most daily ambulatory activities. However, future studies will need to verify this conjecture.

Disadvantages associated with overground calibration, such as lack of a suitable walking area and bad weather, are potential downsides to using overground rather than treadmill calibration. Therefore, despite the relative inaccuracy, the convenience of treadmill calibration may be preferred in some situations. Additionally, it would be more accurate, and therefore appropriate, for treadmill calibrations to be used when patients were to undertake their walking program on a treadmill.

There are several models of Actigraph accelerometers: the 7164 used in this study and new generation models such as the GT1M, GT3X and the GT3X+. Although this study was conducted using an old Actigraph model, there is correspondence between the output of the old and new models. A comparison between the 7164 and 3 versions of the GT1M found no statistically significant differences between outputs during walking and running [35] and, when using the low frequency extension range, the output of the GT3X+ has been shown to be comparable with the 7164 [8, 36]. Also, the mechanisms causing the differences in calibration equations across the treadmill and overground methods do not depend on the monitor and are likely to affect calibration equations obtained using different Actigraph models and other accelerometers in a similar way.

This study was based on a sample of 50 healthy adults. The literature recommends more than 100 participants to obtain reliable estimates of between-individual variability [37]. However, this study aimed at exploring whether the relationships of Actigraph counts with speed and VO_2_ from walking varied across individuals rather than providing reliable estimates of between-individual variability in the population. Future larger-scale studies may want to find an answer to this issue as well as find predictors of between-individual differences in these relationships.

## Conclusions

In conclusion, this study suggests that the proposed track-based individual calibration method can provide accurate and unbiased estimates of free-living walking speed and VO_2_ from walking. It also suggests that treadmill-based calibration should be avoided as it produces calibration equations that tend to substantially overestimate both VO_2_ and speed. Apart from being more valid, the proposed track-based calibration can be undertaken at a lower cost than its treadmill counterpart and may be more acceptable to individuals, especially to those who are not accustomed to walking on a treadmill.
